# Social democracy and social policy in neoliberal times

**DOI:** 10.1177/1440783313492240

**Published:** 2014-12

**Authors:** Christopher Deeming

**Affiliations:** University of Bristol, UK

**Keywords:** class, labour, political sociology, social welfare, sociology, welfare state

## Abstract

This article considers the path of social policy and democracy in Australia and the latest set of welfare reforms under Labor. The reforms can be seen to mark a reaction to the excesses of neoliberal government on the one hand, but they also represent continuity in neoliberal thought and policy on the other. As we shall see, engrained ideas about individualist wage-earning welfare, that were established during the formative years of the 20th century, continue to shape, if not constrain collectivist solutions to some of the inherent social risks faced by Australian citizens today. In this light, efforts to create a welfare state geared towards meeting the needs of ‘hard-working’ Australian families appear much sharper.

With social democracy now in retreat across Europe (Meyer and Rutherford, 2012), this article considers the path of social democratic welfare reform in Australia, and the latest set of social democratic welfare reforms initiated by the Rudd Labor government (2007–10) and the Gillard Labor government (2010-13). The reference to ‘social democracy’, itself a contested term (Glyn, 2001), indicates the broad reformist tradition, and social policy programmes, of the Australian Labor Party in power. This article begins with a brief look at the original and formative accounts of social democratic welfare in Australia as portrayed by Francis Castles (1985), for example, before reflecting on the neoliberal turn in social policy and ‘economic rationalism’ during the 1980s and 1990s in the following section. Attention turns to the distinctiveness, and thus positioning, of the Australian model of welfare in the debates about the different worlds of welfare. In the third section, we focus on the revival of ‘social democracy’ and the ‘social investment state’ in the early part of the 21st century and we draw upon the latest cross-national trend data and economic statistics to help situate developments in welfare policy. Finally, we reflect on the path of welfare reform and we consider the extent to which Australian social policy could be considered distinctive in the ‘neoliberal’ age of capitalism (Dean, 2014).

In this article, we are interested in comparing the architecture of the Australian welfare state against other leading economies. Throughout, the charts rely on data from the OECD (Organisation for Economic Co-operation and Development) (2007, 2008, 2010);^1^ the cross-national comparisons follow the now familiar pattern in this field (shown in Table 1). We should observe that all data are prone to measurement error and data from the OECD are unlikely to be exceptional in this regard. OECD data, however, tend to be of a high quality and are generally considered robust for drawing international comparisons on economic statistics; often they are the best we have available to assist with comparative policy research (Giovannini, 2008).

**Table 1.** Worlds of welfare and families of nations.

	Liberal	Conservative	Social Democratic	Radical
Esping-Andersen (1990)^a^	Australia	Finland	Austria	
	Canada	France	Belgium	
	Ireland	Germany	Denmark	
	New Zealand	Italy	Netherlands	
	United Kingdom	Japan	Norway	
	United States	[Switzerland]	Sweden	
	[Switzerland]			

Castles and Mitchell (1993)^b^	Ireland	Germany	Belgium	Australia
	Japan	Italy	Denmark	New Zealand
	Switzerland	Netherlands	Norway	United Kingdom
	United States		Sweden	

a Switzerland was cross-classified by Esping-Andersen, being Conservative on his index of decommodification and Liberal on his index of social stratification.

b The four countries of Austria, Canada, Finland and France were not accommodated within Castles’ and Mitchell’s classification.

## The constitution of the workers’ ‘social investment’ state, 1890 -1980

In Australia, the original emphasis of social policy was placed on the protection of male breadwinners and their families, for which Castles (1985) coined the term ‘wage earners’ welfare state’. Social democratic efforts were directed at securing acceptable conditions of work, including legislative measures to pay male workers a fair and reasonable ‘family wage’. The social policy framework stood in sharp contrast to the institutional arrangements of state welfare in the Scandinavian countries of Denmark, Norway and Sweden (Castles, 1978; Korpi, 1978). There redistributive social policies from the 1930s were designed to promote equality as a citizen’s right. In Australia, by contrast, redistributive efforts were achieved using the instruments of wage regulation rather than public expenditure in traditional areas of welfare, as seen in the Scandinavian countries.

Australia’s ‘social investment state’ played a key role in the economy at the turn of the 19th century, as Smyth (2004) observes, with extensive government investment in capital infrastructure, utilities and state-enterprises. Industrial regulation provided a formative pathway for social policy in Australia and the orientation towards a ‘welfare society’, rather than a ‘welfare state’. Minimum wage laws were introduced in South Australia in 1894 and then, in 1907, a ‘basic wage’ judgment, the ‘Harvester Judgment’ (Higgins, 1922), gave courts in Australia the power to determine acceptable working conditions including (minimum) living wages for families, based upon the needs of the average family (consisting of a man, his wife and three children).^2^ Therefore, because the distinctive focus of Australian social policy was oriented towards ‘pre-distribution’ via regulation of the wage relationship, Castles pursued the idea of the wage-earning model of welfare in his comparative study of social policy (see Deeming 2013 for a review of this work and the New Zealand policy context). The central argument here makes working-class politics the (over-)determining factor in shaping social policy legislation in Australia, but critics argue this account overlooks historical contingency and some of the wider social and political factors at play (Castles, 1997; Watts, 1997). Nevertheless, the labour movement and trade unionists had succeeded in their fight for just wage prescriptions defined by the state; other employment-related benefits followed, means-tested occupational and disability pensions in 1909, and unemployment and sickness benefits in 1944 paid from general taxation revenues under the Curtin-led Labor government (1941–5) (Castles, 1992). From 1945, Labor economic policy followed Keynesian principles, the Keynesian ‘economic state’. Thus, the value of investment was to be moderated by the government in order to maintain the economy in a full state of employment (Labor policy from 1945) (Smyth, 1994).

In Australia, there was little political appetite for social insurance or an expanded system of welfare paid out of general taxation because wage control was the means for securing needs-based welfare, or welfare ‘by other means’ (Castles, 1989).^3^ In the ‘New World’ those workers who owned their own homes were able to maintain a decent standard of living for their families (Dalton, 2010). Australia, for instance, had a home ownership rate near 50 percent in the early 1900s, reaching 70 percent by 1960. For anyone outside the labour market, however, the standard of living was markedly different. Lone or sole parents, those with disability, and Indigenous Australians were particularly vulnerable to poverty. Liberals objected to the ‘family wage’ policy and attempted to undermine it. They believed the pay floor distorted the labour market, but they also saw a moral hazard: the wage policy clearly favoured male workers without dependent children and family responsibilities. Facing union demands to increase wages in 1941, for instance, the Menzies government introduced Child Endowment throughout Australia (Bessant et al., 2006).^4^

**Figure 1.**
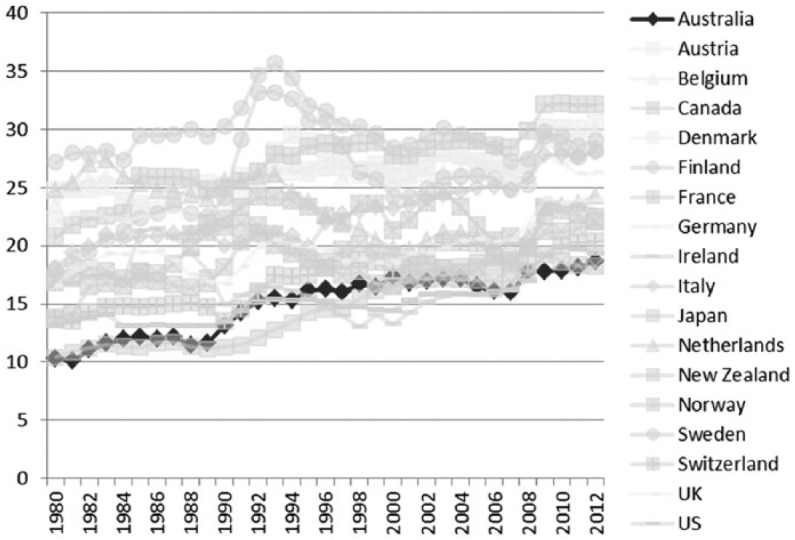
Public expenditure on social welfare in OECD countries (in percentage of GDP), 1980–2012.

The workers’ welfare state thesis was supported by early comparative social statistics, which begin to emerge with the founding of the Organisation for Economic Co-operation and Development in 1960, in Paris (OECD, 1972). During the 1950s and 1960s, public spending on social welfare can be seen trailing behind that of most of the other OECD countries. For example, total expenditure on social security was around 5 percent of GDP per annum; the OECD average was 8 percent. This picture hardly changed in the subsequent decades of the 1970s and 1980s, despite efforts by the Whitlam Labor government (1972–5) to move Australia towards a more universal ‘welfare state’ model.^5^ In Australia, the total welfare bill averaged 13 percent of GDP each year during the 1970s, compared with an OECD average of 19 percent. By the mid 1980s, when Castles published his comparative analysis of social policy, welfare spending in Australia trailed the OECD average by eight percentage points. Within the OECD group, we find Australia at the bottom of the pack (Figure 1), only Japan was spending less each year on welfare services. This evidence helped support the workers’ welfare state thesis: Australia appeared a conspicuous welfare laggard largely because social policy efforts for much of the 20th century had been geared towards ensuring that work paid a sufficient wage to cover the costs of keeping a family, thus male workforce participation was the primary way that family households produced welfare. Australia did not have a Keynesian-style welfare state and, as a result, social citizenship entitlements remained weak (Mitchell, 2004).

## Economic rationalism in the 1980s and 1990s

**Figure 2.**
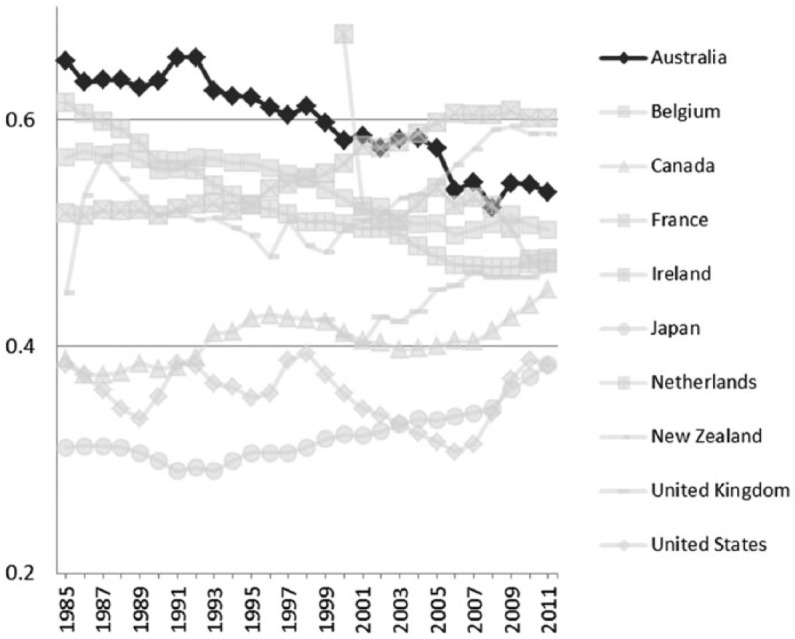
Minimum wages relative to median wages of full-time workers in OECD countries, 1985–2011.

**Figure 3.**
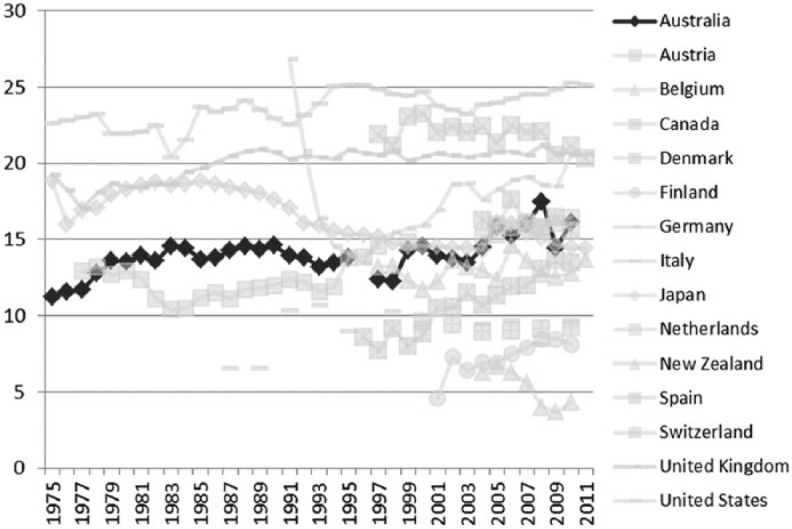
Trends in the incidence of low pay in OECD countries (percentage of full-time workers earning less than two-thirds of full-time median wages), 1975–2011.

During the 1980s and 1990s, Australia, like most other developed countries, experienced a neoliberal backlash against state intervention in the market. Globalizing forces, in the form of increasing economic integration and world trade, significantly altered the shape of the Australian economy and the nature of society. The Hawke Labor government (1983–91) shifted to the right, adopting free-market reforms. By the late 1980s the goal of full employment was a distant dream and the Accord between Labor and the unions had ceased to be an effective policy instrument of wage control (Quiggin, 1998).^6^ Some of the effects of deregulation are clearly evident in the comparative data. Minimum wage rates relative to average earnings are in decline (Figure 2), and the incidence of low pay has steadily increased (Figure 3). Australia became a much more unequal society (Saunders, 1994).^7^ However, the growth in female employment over the period helped to ease the pressure on living standards for dual-earner families (Watts, 1993). Australia was slowly shifting the model of social policy from being one which confers social citizenship on women via a male breadwinner towards a model that addresses social rights on a more individual basis.

**Figure 4.**
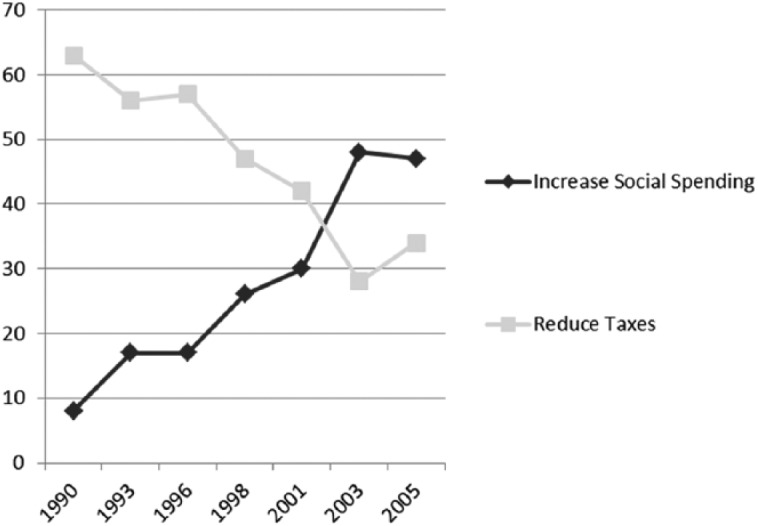
Public attitudes to tax and welfare in Australia (percent of Australian electorate), 1990–2005.

With growing unemployment and Australia in recession in the late 1980s and early 1990s, income tax rates were cut, a popular move with the electorate at the start of the 1990s, as shown in Figure 4. The goals of social policy were redefined, as social security became more targeted (Saunders, 1991). Means-tested allowances focused welfare assistance, but clearly failed those families most in need (Veit-Wilson, 1998). In the absence of a human right to an adequate income for all citizens, it may not be surprising to find the public commitment to social welfare in Australia trailing other OECD countries at the close of the last century. Social welfare expenditure in Australia increased by about 70 percent over this period helped by the introduction of Family Allowance Supplements in 1987, as shown in Figure 1. Despite the increases, overall spending on social welfare was 20 percent below the OECD average at the close of the 20th century, putting Australia at the bottom of the comparative OECD group (Figure 1).

The concept of ‘economic rationalism’, placing the market before state and society (Pusey, 1991), that began in the 1980s under the Hawke Labor government, became more accepted in the 1990s under the Keating Labor government (1991–6) and the Howard-led Liberal–National coalition government (1996–2007). This ‘refurbishment’ of welfare policy included the introduction in 1996 of a rebate on private health insurance that threatened the public health care system, and industrial relations reforms that undermined the long-established system of independent arbitration. Further deregulation of the wage structure, marked by Work Choices (the Workplace Relations Amendment Act 2005), saw the removal of employment laws relating to unfair dismissals. The Liberals were looking to increase national economic performance and, following standard neoliberal philosophy, they firmly believed that a more flexible labour market was necessary to achieve this outcome. Critics maintained that the laws stripped away basic employee rights and were fundamentally unfair. Certainly, the principal thrust of the Work Choices legislation was to individualize employment relations, with the effect of marginalizing trade unions and industrial tribunals.

**Figure 5.**
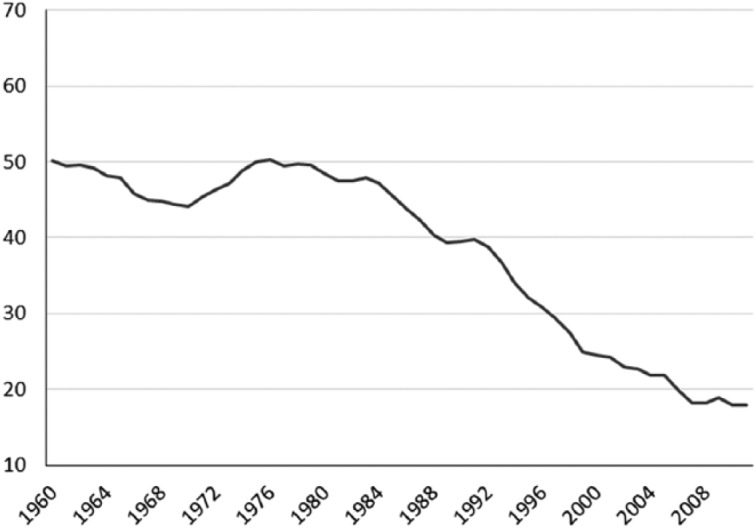
Trade union density in Australia (percentage of employees), 1960–2011^a^. ^a^Trade union density corresponds to the ratio of wage- and salary-earners who are trade union members, divided by the total number of wage- and salary-earners.

Clearly, there has been a significant decline in union membership over this period (Figure 5), raising important questions about the relevance of trade unions for social democracy in the Australian context. Social attitude surveys of the time suggest that these reforms were not altogether popular with the electorate: 40 percent of Australians disapproved of the labour market reforms and about 80 percent believed that industrial legislation was needed to protect workers from unfair dismissals (Van Wanrooy, 2007). Many Australians also believed that widening inequality was not good for the country; and a significant minority (40 percent) wished to see greater redistribution (Wilson and Meagher, 2007).

Academics and policymakers have analysed the impacts of these policies on the Australian model of welfare. This analysis needs to ‘locate’ this model of welfare within existing comparative policy frameworks. This task continues to provoke controversy; with much of the dispute focusing on the work–welfare nexus in modern economies. For some observers, the architecture of the Australian welfare state was characteristically liberal in design, recognizable by the residual system of social security based on means-tested social assistance schemes and targeted income support benefits. Certainly, Esping-Andersen (1990), in his original work on the *Three Worlds of Welfare Capitalism*, argued that Australia belonged to the liberal world of welfare capitalism, along with other Protestant, Anglo-Saxon countries such as the UK, the United States, New Zealand and Canada (Table 1). Over the years, many have given their support to this framework and the positioning of Australia within it (e.g., Leibfried, 1993; Saunders and Pinyopusarerk, 2001; Siaroff, 1994). By contrast, Francis Castles provided one of the earliest challenges, at least in part, to Esping-Andersen’s welfare typology. According to Castles and Mitchell (1993), Australia and New Zealand were ‘other worldly’, being significant social policy innovators (along with the UK). They belonged to a ‘fourth world’ of welfare, as shown in Table 1.

Somewhat counter-intuitive perhaps is the ‘radical’ depiction of Australian social policy within the alternative ‘families of nations’ classification posited by Castles and Mitchell (1993). However, the regulation of the labour market and the early minimum wage laws helped to provide the justification, along with government welfare expenditure levels and the progressive system of income tax. Those claiming a ‘fourth world’ drew a firm distinction between the targeted income protection scheme found in Australia and the basic model of welfare services found in the liberal world (e.g. Castles, 1993; Huber and Stephens, 2001; Korpi and Palme, 1998; Shalev, 2007). Surprisingly, perhaps, Esping-Andersen was later persuaded by Australian exceptionalism (Esping-Andersen, 1997), while Castles (2001) saw Australia as being drawn into the liberal family of nations. Castles claimed, erroneously perhaps, that Labor had also abandoned its commitment to wage-earners as the effects of labour market deregulation were felt. The notion of decommodification is inherently male, however, and women’s entitlements (whether through paid work or social security) have been found by feminist scholars to be more variable across the Breadwinner Model states (O’Connor et al., 1999).^8^

## Twenty-first-century ‘social investment’

According to theories of path dependency in social policy, welfare states create vested interests and economic incentives that militate against path-departing processes (Castles, 2010). A sudden crisis, however, can affect the character of social policy and welfare state development. It was, for example, the oil crises of the 1970s that helped to usher in neoliberal reforms in Australia, while the recent global financial crisis – fuelled by changing public sentiments about the good society (Figure 4) – has helped to instigate the new era of social democracy (Saunders and Deeming, 2011). The Rudd Labor government was elected in 2007 and immediately found itself grappling with the global financial downturn. Like many other governments around the world, it reverted to Keynesian social investment policies in order to stimulate the economy in the face of recession. Kevin Rudd and Julia Gillard clearly set themselves apart from New Right ideology, arguing instead for social democratic values and principles of social inclusion (Australian Government, 2009a). Before taking office, Kevin Rudd (2006) had attacked John Howard for abandoning the basic principles of social justice in Australia that had been embraced by the centre-right liberals and conservatives over much of the previous century.

**Figure 6.**
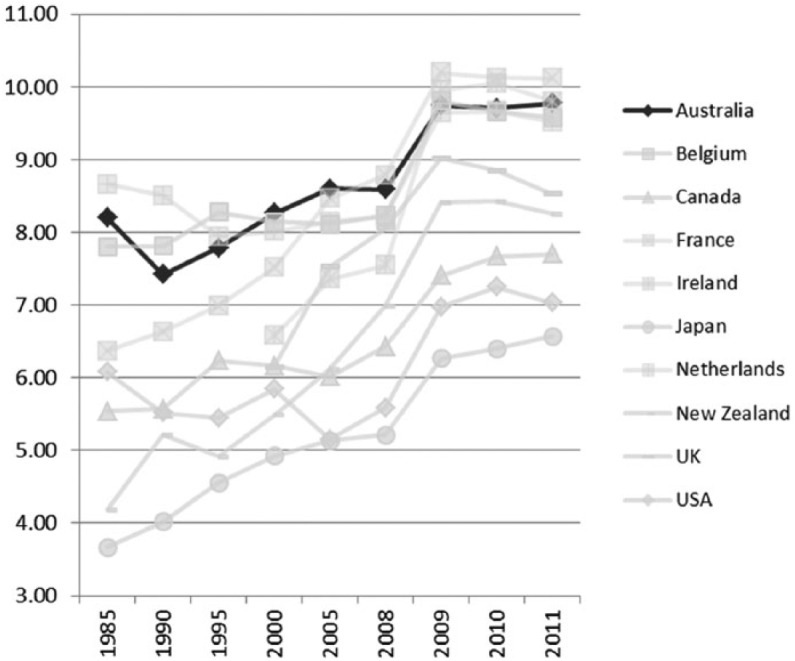
Minimum wage rates in OECD countries (hourly minimum wages US PPP), 1985–2011.

The priority for the Labor government has been to strengthen the system of welfare for ‘working families’; this term, which has positive connotations, was extensively used by Kevin Rudd and Julia Gillard during the 2007 federal election (Maher, 2008). Successive Labor budgets over the last four years (2008–12), for instance, have been mildly progressive, intent on improving living standards for ‘hard-working families’ (Australian Government, 2008). The government has provided tax relief for low-income households and child care tax rebates for working families to the tune of $47 billion (Australian Government, 2011). The Fair Work Act (2009) reversed the unpopular labour market polices of the 1990s and provides workers with a safety net of employment conditions. In particular, Labor restored to Australian workers the legal right to appeal against harsh or unfair dismissals from their place of work – a right that had been rescinded by Work Choices. Once again, collective bargaining is encouraged and wages are to reflect relative living standards and the needs of the low paid, as well as the potential impact of changes on the labour market and unemployment levels. Despite falls in the late 1980s and early 1990s, minimum wage protection levels in Australia have been increasing, offering greater protection to low-paid workers (Figure 6).

Targeted tax cuts for ‘working families’ and the Fair Work initiative, which reinstates the notion of appropriate minimum wage rates on social justice grounds, could, if we accept the idea of a policy pathway, be seen to mark a return to more familiar territory for Australian social policy. However, the contemporary social democratic discourse is not only about protecting low-income working families from the perils of the free market; social democratic principles and the social policy framework, in the form of tax breaks, such as the Low Income Tax Offset (LITO), the Child Care Rebate and Family Tax Benefit (FTB), have also now been extended to help meet the needs of more affluent working families on middle incomes (Australian Government, 2011). Thus, ‘middle-class’ families have been brought into the ‘fiscal’ welfare system with tax exemptions and rebates, which, according to some commentators, has created a ‘dual welfare state’ in Australia (e.g. Stebbing and Spies-Butcher, 2010).

**Figure 7.**
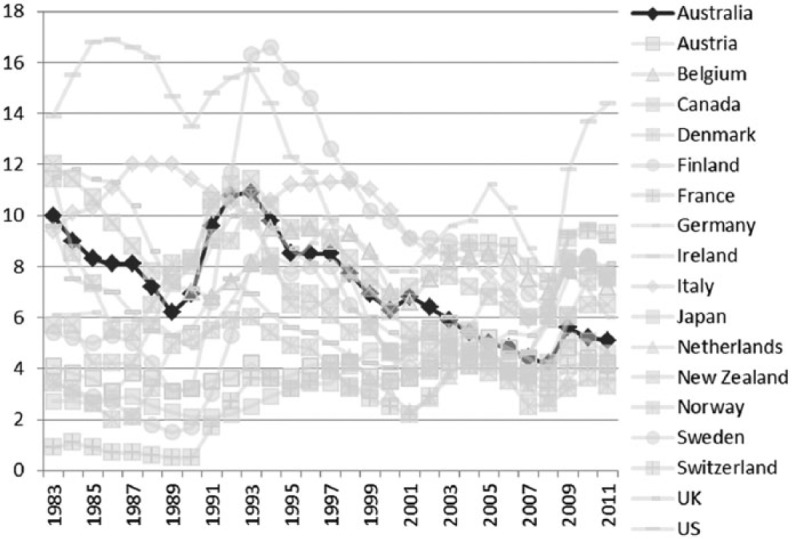
Unemployment rates in OECD countries (percent), 1983–2011^a^. ^a^The unemployment rate is the ratio of number of persons unemployed and the number of persons in the labour force.

Like its international counterparts, the Australian Labor Party is now content to pursue the (diminished) goal of full employability (as opposed to full employment), with mutual obligations and increased investment in training provision being key to helping the unemployed back into the labour force (Australian Government, 2010a). The very notion of ‘hard-working Australian families’, the preferred expression of Julia Gillard (2010) in her recent Address to the Nation, is not just a rhetorical device aimed at a political constituency – those on low-to-middle incomes – it also articulates a range of normative assumptions about the way in which life should be lived in Australia in the 21st century. Work is seen as the best form of welfare, not only because work pays better than welfare but also because it promotes well-being for families. Therefore, one of the key goals of Australian social policy, particularly with the introduction of new regulatory work-based welfare measures, has been to help people get off welfare benefits and into work. Benefit claimants are now finding new conditions and forms of enforcement attached to social security payments: being available for work and actively seeking employment and being able to demonstrate this, for example, are minimum codes of conduct. The role of the state is to facilitate ‘activation’, with new training programmes to improve skills and increase job readiness. For the goal of ‘workfare’, or ‘welfare-to-work’ as it is sometimes known, is not just to reduce unemployment, but also to tackle the wider problem of worklessness. Long-term unemployment can deskill. Training unemployed people in the skills required by employers is therefore part of the supply-side approach adopted by the Labor government to overcome shortfalls in the economy. The more compassionate approach to economy and society appears to be helping to keep unemployment rates below the OECD group average (Figure 7); the number of people out of work now appears to be falling again following the sharp rise resulting from the economic downturn in 2008.

**Figure 8.**
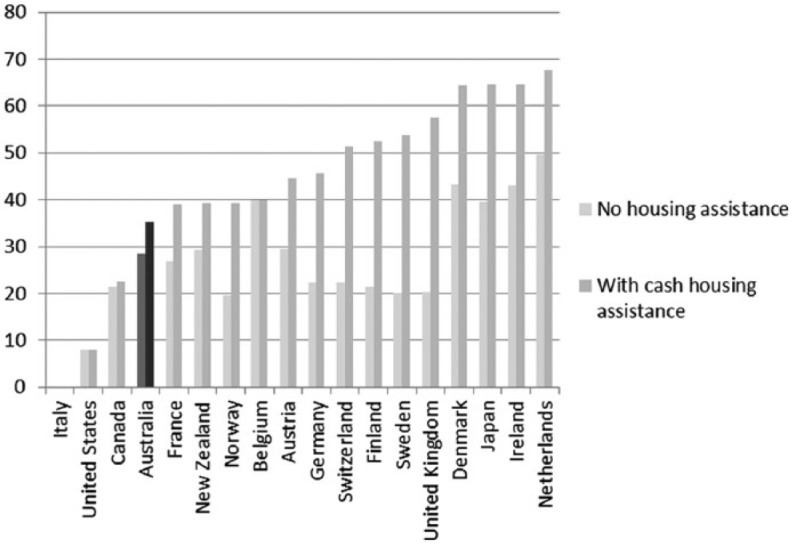
Income levels provided by minimum-income benefits for adults in OECD countries (net income value in percent of median equivalized household incomes), 2010.

**Figure 9.**
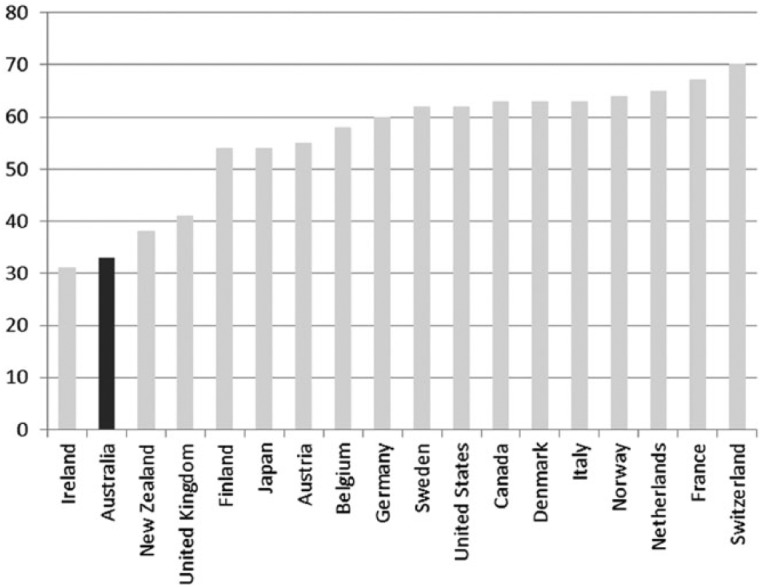
Unemployment benefit replacement rates for adults in OECD countries (% of average wage levels in 2005).

**Figure 10.**
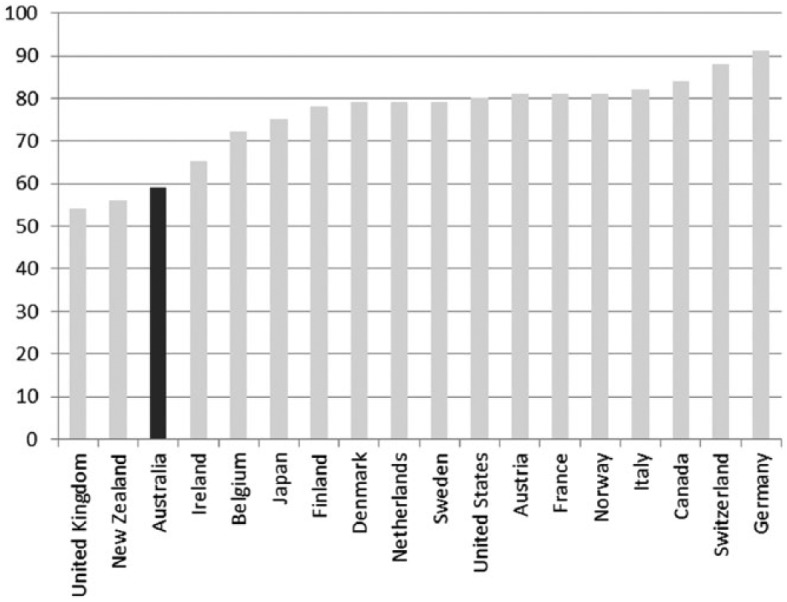
Unemployment benefit replacement rates for families with two children in OECD countries ( percent of average wage levels in 2005).

A long-standing concern in Australian social policy has centred on the welfare of those not in paid employment. Social policy scholars continue to question the adequacy of the social security system for those not in paid work (Saunders, 2011; Soldatic and Pini, 2012). Decommodification levels in Australia are amongst the lowest in the Western world. Thus, Australians appear to accept the risk of a relatively low standard of living and poverty if they are unable to work or become unemployed (OECD, 2007, 2011a). Minimum-income benefit levels for adults in Australia are below the international poverty line of 60 percent median equivalized household income; calculations in Figure 8 are shown with housing benefits and excluding them. Benefit replacement rates, paid in the initial phase of unemployment, are also low compared to other advanced economies. For an unemployed adult, benefit replacement rates amount to about one third of average wages; only unemployed adults in Ireland receive less (Figure 9). The situation for families is not much better with benefit replacement rates below 60 percent of average wages. We see from Figure 10 that only unemployed families in New Zealand and the UK are worse off than those in Australia.

**Figure 11.**
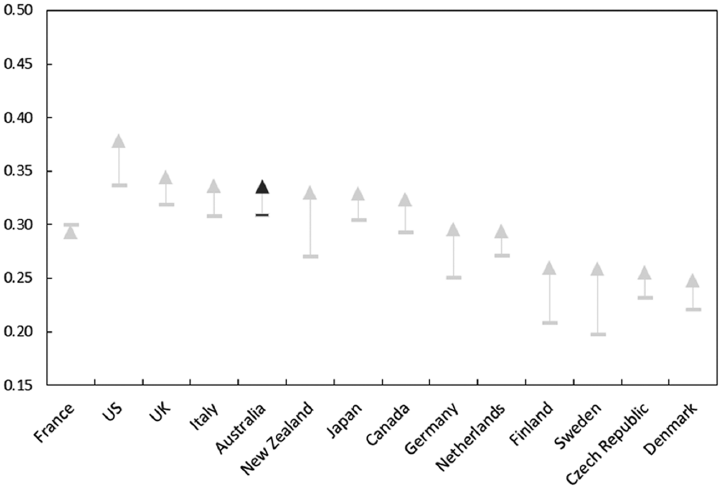
Income inequality in selected OECD countries (Gini coefficients)^a^. ^a^Gini coefficients of income inequality. Arrows indicate the change and direction of income inequality from the mid 1980s to the late 2000s.

**Figure 12.**
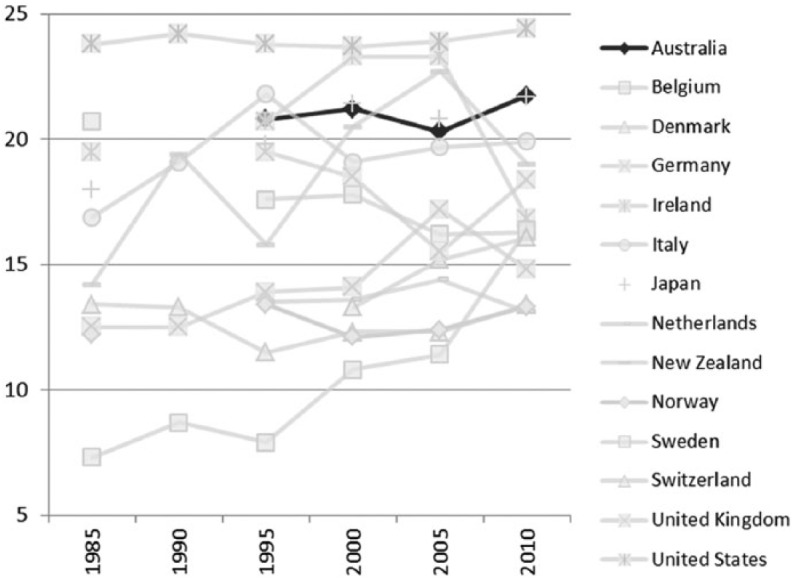
Poverty rates in OECD countries (percent of the population living below the international income poverty threshold), 1985–2010^a^. ^a^Poverty defined as 60 percent of the median equivalized household incomes.

Income inequality has increased significantly since the mid-1980s (Figure 11). Over 20 percent of Australians live in poverty, placing the country above most of the other advanced economies on this scale (Figure 12). Only the US and Ireland have higher rates of income poverty. The risk of poverty is not evenly distributed across Australian society. Those who are unemployed and jobless, adults with disability, Indigenous Australians and single parents face a higher risk of poverty. Saunders (2011), for example, argues that it would require only a modest increase in social security payments to shift Australia a long way up the international poverty league table, but work incentives take priority over standards of adequacy in Australian social security policy (Deeming, 2011).

**Figure 13.**
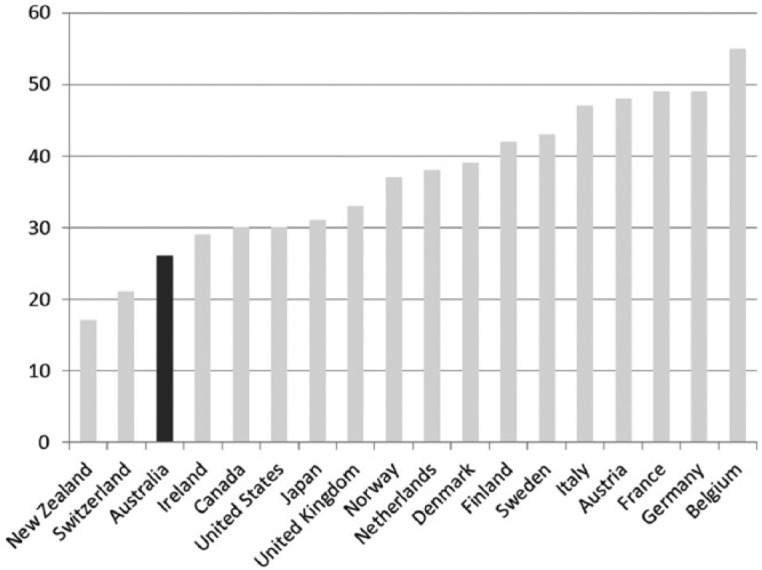
The tax burden on waged income in OECD countries (total tax wedge %), 2010^a^. ^a^Average personal income tax and social security contribution rates for adults at % of average wages.

Instead, Labor appears content to preserve the current system of redistribution. Australia’s tax-transfer system is progressive by international standards, and the benefits system remains one of the most targeted in the developed world (OECD, 2008, 2010). As Whiteford (2010) observes, the Australian system plays ‘Robin Hood’ by targeting assistance to lower-income families using high means-test thresholds to ensure payments to better-off households are minimised and, through low levels of direct taxes, to ensure that very little of the assistance directed to low-income families is clawed back. The present tax and transfer system, which has long favoured market freedom and individual opportunity, serves Australia well according to the recent independent review of taxation policy (Henry Review, 2009). Only Switzerland and New Zealand appear to have lower tax rates than Australia (Figure 13).

There are strong continuities with but also critical differences from the ‘neoliberal’ agenda (Dean, 2014), particularly with the role of the ‘enabling’ or ‘social investment state’ taking greater responsibility for workforce participation, for skills, training and employment strategies. Keynesian principles of social investment in human capital and infrastructure are being revived (Australian Government, 2009a). The traditional social democratic concern with social justice remains, but the relationship between the state and recipients of welfare has been recast in a new contract of ‘rights and responsibilities’, framed by new thinking about inclusive economic growth (Smyth and Buchanan, 2013). Policymakers now firmly believe that all adults who are able to work should be in the labour force, thus increasing the centrality of paid work to the securing and production of family welfare. The radical neoliberal programme of welfare reform was back-tracked by Labor in favour of a return to ‘social investment’, but only to a degree. The fiscal market and mixed economy changes have endured, as have the competitive and quasi-market approach to organizing and delivering public services (Considine et al., 2011). Thus far, in the wake of the financial crises and with the continued threat of recession, the priority has been to maintain a grip on welfare spending (Figure 1).

**Figure 14.**
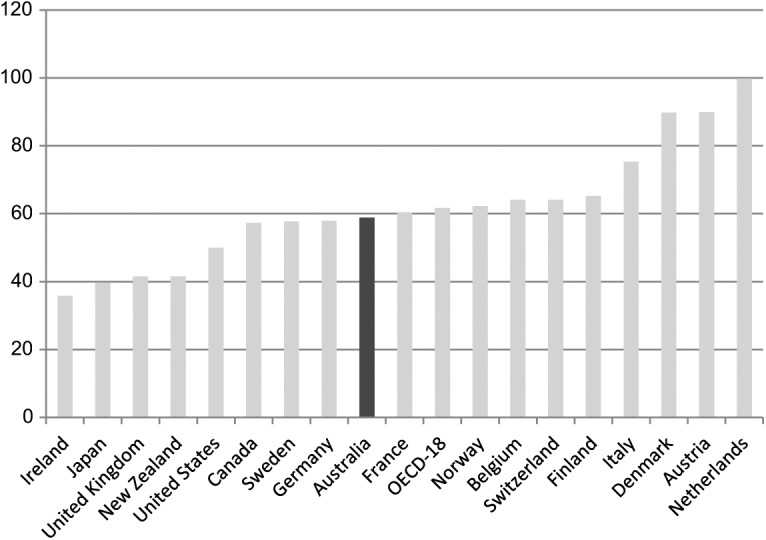
Net pension replacement rates in OECD countries (percent of average earnings in 2011).

Following the Harmer Review (2009), however, Labor raised the minimum-income floor for old age pensioners to 28 percent of average weekly male earnings. Figure 14 shows pension replacement rates in Australia for average earners at 59 percent of average wages, slightly below the study group average (OECD, 2011b). Recent reform discussion in Australia has focused on better integration of the tax and benefit arrangements in the superannuation system. From July 2013, the Superannuation Guarantee (SG) rate will start to increase from 9 to 12 percent of wages, which will deliver much better outcomes for 8.4 million Australians. While the Low Income Superannuation Contribution (LISC) is a new contribution tax rebate specifically designed to benefit some three-and-a-half million low-income workers. The present Labor administration has also introduced the national Paid Parental Leave scheme in 2011 and DisabilityCare Australia from 2013, a healthcare program initiated by the Australian government, with $19.3 billion over seven years to support those people who have a significant and permanent disability (Australian Government, 2013).

**Figure 15.**
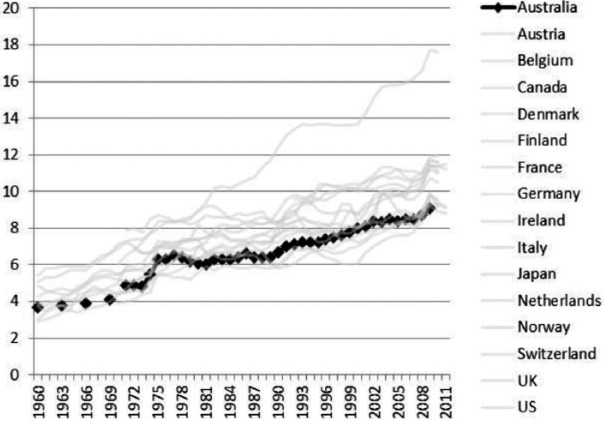
Public expenditure on health care in OECD countries (percentage of GDP), 1980–2008.

Another key social democratic reform has been to strengthen the Australian national health service, which has been fragmented on State lines (NHHRC Review, 2009). The Labor government, with the backing of State premiers and ministers, is intent on reform. The federal government will now be responsible for providing much of the funding for public hospitals and primary care services (National Health and Hospitals Network Act 2011). Historically, public expenditure on health care has been relatively low compared to other leading economies; it currently stands at about 6 percent of GDP and trails behind the OECD group, as shown in Figure 15. Although many Australians are covered by private health insurance (almost 45 percent), survey data also suggest that there is popular support for the public health system and Medicare, including support for higher taxation to help pay for improvements (Wilson et al., 2005).^9^ Polling of Australian social attitudes suggests a distinct dual trend: increasing public support for investment in welfare services, on the one hand, and a decline in public support for further tax cuts, on the other (although the polling evidence shown in Figure 4 pre-dates the global financial crises).

**Figure 16.**
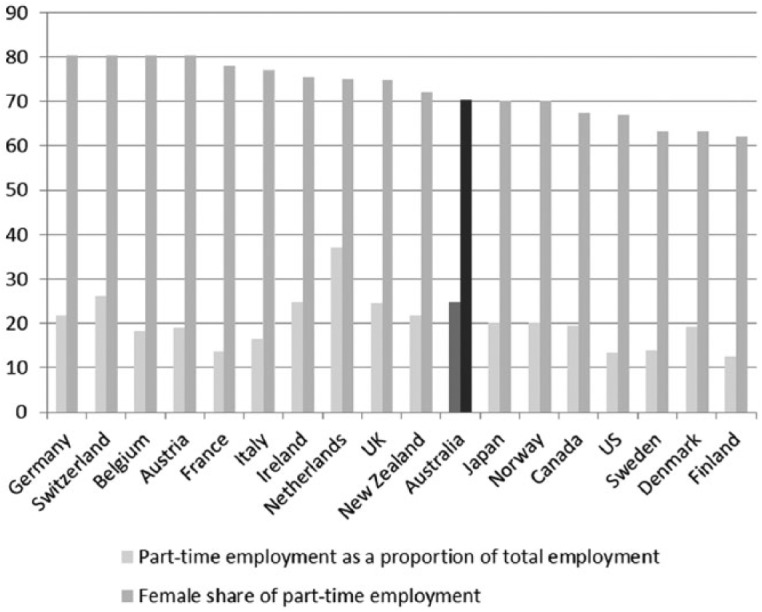
Female share of part-time work in OECD countries (percent of all part-time employment), plus part-time work as a percentage of total employment, 2010.

In all OECD countries there has been a shift towards temporary labour; this is particularly evident in Australian context with growing numbers of fixed-term and part-time employment contracts (Figure 16). Labour market ‘flexibility’ is now part of global capitalism and is the result of the neoliberal economic policies implemented in most countries in the last 30 years. However, we see in Figure 15 that women appear to be much more ‘flexible’ workers than men. In Australia, the share of part-time employment among female workers currently stands at 70 percent. Part-time work and fixed-term contracts help to explain the inferior labour market position of women, as Lewis (2009) observes. They are also disproportionately responsible for care work.

**Figure 17.**
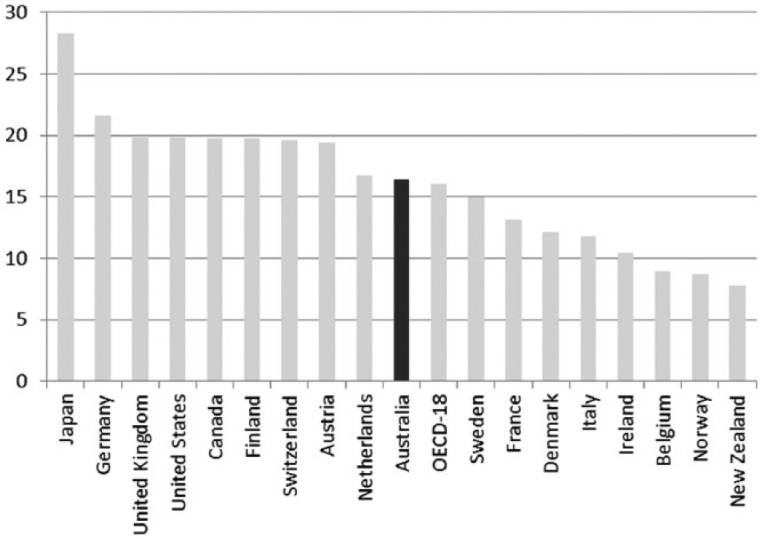
Gender wage gap in OECD countries (percentage difference between median earnings of men and women), 2009^a^. ^a^The gender wage gap is unadjusted and is calculated as the difference between median earnings of men and women relative to median earnings of men.

Social inclusion has been at the centre of the Australian social policy agenda since 2007 (Australian Government, 2009b, 2010b). And, while much has been achieved, it remains unclear whether the new policy focus has made a substantive difference in practice (as Saunders (forthcoming) argues) to what would have been achieved by any new centre-left government coming to office after a decade of neoliberalism. The government estimates that 5 percent of working-age Australians are at risk of being left behind; they continue to experience multiple and entrenched social disadvantage. Moreover, gender and ethnic inequalities stubbornly persist in Australian society and pose a major challenge for policymakers (Office for Women, 2009). Australia is a long way from gender pay parity – a key measure of fairness – despite the policy of ‘equal pay for equal work’ (OECD, n.d.). The average difference between median wages for men and women is more than 16 percent, placing Australia above the OECD group average ranking (Figure 17). Progress on tackling Indigenous peoples’ disadvantage remains slow despite a range of policy initiatives such as ‘Closing the Gap’, designed to create a more inclusive Australia (AIHW/AIFS, 2012; Australian Government, 2009c; Gray and Hunter, 2005): the unemployment rate for people of Aboriginal and Torres Strait Islander origin in Australia, at 20 percent, is over three times the national average. The re-engineering of welfare policy by the latest Labor governments is arguably struggling to tackle the deeply entrenched gender, cultural and ethnic inequalities. Drawing together the continuities and breaks in social policy, as implemented by the Labor party, we find ongoing structural changes in society, a result of global transformations, and the shift toward temporary labour, resulting in persistent poverty and evidence of growing inequality.

## Conclusions

Two conclusions are drawn on the Australian social policy experience. First, in view of recent reforms, and socio-economic transformations, there has been much more than a rhetorical switch from the male breadwinner model of welfare to a new social democratic investment model of ‘welfare society’ geared towards meeting the needs of ‘hard-working families’, that is, the dual-earner families on low-to-middle incomes, who now receive ‘welfare-in-work’. Here we might wish to draw attention to the role of politics in the policy process, determining social policy outcomes. The old male ‘breadwinner, female home-maker family model of welfare’ grew out of class struggle and conflict, and, in turn, it is class politics that continue to shape, if not constrain, today’s welfare settlement. In the battle over the political middle ground, middle-class families have been brought into the social security system. Australia is not alone in this respect; many other countries have similar systems that churn tax and welfare using tax rebates and tax credits, grants and wage supplements. In the USA and the UK, for instance, the battle is also on for the hearts and minds of ‘hard-working families’, that is, those on low-to-middle incomes (Parker, 2013; Whittaker and Bailey, 2012). The British Labour government (1997–2010) sought to create a system of welfare suited to their needs during its term in office, and the Left remains committed to this cause. The new Conservative–Liberal Democrat coalition government is also appealing to this constituency, the hard-workers or ‘strivers’. The appeals are becoming increasingly fraught as debates over fairness and social justice rage, heightened by the new politics of austerity (Farnsworth, 2011). Second, our analysis of the economic statistics and welfare trend data reveals important continuities in Australian social policy. Protection against social risks, unemployment and sickness, remains very much an individual responsibility. Although DisabilityCare Australia, which has come into play through the mediating influence of shifting social attitudes (Figure 4), has the potential to transform disability services. Raising taxation levels, however, to pay for universal social services continues to be politically charged, particularly where a deeply held belief prevails that hard-working Australian families are bound by moral obligation to look after themselves. Thus, public expenditure on social welfare has been contained at relatively low levels.

Paradoxically, in the new Age of Austerity, Australian social policy may start to look less distinctive as the other advanced economies cut back on welfare spending, and in so doing move further towards the privatization and marketization of risk in society (Adema et al., 2011; Immervoll, 2010). Independent intergovernmental organizations such as the IMF (2012) and OECD (2013) warn of further rounds of austerity. Particularly in Europe, there is widespread concern that governments are spending too much on their welfare programmes amid severe economic and political crisis. After a second generation of austerity measures across the developed world, the Australian character of social policy (the ‘Australian way’ (Smyth and Cass, 1998)) may not appear so distinctive at all.

## Notes

1. Data quoted in the figures are taken from the OECD Social Expenditure Database (SOCX), OECD Tax Database, OECD Employment and Labour Markets, OECD Health Data, OECD Database on Trade Unions, OECD Income Distribution Database, OECD Income Distribution and Poverty Database, all available via OECD.Stat (http://stats.oecd.org/Index.aspx).

2. In Australia, there is no automatic link between the federal minimum wage and wages set under State awards. Although in practice, all States except for Tasmania have applied the federal minimum wage to workers under State awards since 1997. This is different to the UK, which has had a national minimum wage since 1999.

3. The Lyons’ government had attempted to introduce a comprehensive welfare insurance scheme in 1938 – covering health and pensions – but the scheme was not implemented. Contributory insurance was opposed by Labor (as well as other vested interests including doctors and charities), as it did not cover unemployment benefits to be funded from consolidated tax revenue.

4. From July 1941 Child Endowment was paid directly to the mother, at a flat rate of five shillings per week for each child after the first under the age of 16 years. Child Endowment was renamed Family Allowance in 1976.

5. The creation of a national health service, for example, and the extension of the aged pension to everyone over 70 years of age were among the initiatives.

6. The Prices and Incomes Accord on Economic and Social Policy between the Australian Labor Party and the Australian Council of Trade Unions (ACTU) ushered in a new phase in industrial relations with the election of the Hawke Labor government in February 1983. Unions agreed to modest growth in real wages and the government pledged to minimize inflation. It was also through the Accord that the Keating Labor government introduced Superannuation Guarantee Charge (SGC), requiring employers to set aside contributions for their employees in superannuation funds.

7. The Gini coefficient, for example, rose from 0.27 in the early 1980s to 0.31 in the mid 1990s. The Gini coefficient varies between zero and one, if everyone had exactly the same income then the Gini coefficient would be zero (i.e. perfect equality), and if one household had all the income, then the Gini coefficient would be one (i.e. complete inequality).

8. Unlike ‘decommodification’, ‘defamilisation’ is a concept often used to refer to the degree to which individual adults can maintain a socially acceptable standard of living, without being forced to marry or enter into other family relationships. O’Connor et al. prefer the notion of ‘autonomy’ to ‘defamilisation’ however.

9. According to evidence from the Australian Survey of Social Attitudes (AuSSA), 52 percent of the population were prepared to pay more for public health services and Medicare in 2003.
